# Learning Evaluation: blending quality improvement and implementation research methods to study healthcare innovations

**DOI:** 10.1186/s13012-015-0219-z

**Published:** 2015-03-10

**Authors:** Bijal A Balasubramanian, Deborah J Cohen, Melinda M Davis, Rose Gunn, L Miriam Dickinson, William L Miller, Benjamin F Crabtree, Kurt C Stange

**Affiliations:** Department of Epidemiology, Human Genetics, and Environmental Sciences, University of Texas Health Science Center Houston School of Public Health, Dallas Regional Campus, 5323 Harry Hines Blvd, V8.112, Dallas, TX 75390 USA; Harold Simmons Comprehensive Cancer Center, UT Southwestern Medical Center, Dallas, TX USA; Department of Family Medicine, Oregon Health & Science University, Portland, OR USA; Department of Family Medicine, University of Colorado School of Medicine, Aurora, CO USA; Department of Family Medicine, Lehigh Valley Health Network, Allentown, PA USA; Department of Family Medicine and Community Health, Robert Wood Johnson Medical School, Rutgers University, New Brunswick, NJ USA; Departments of Family Medicine and Community Health, Epidemiology and Biostatistics, and Sociology, Case Western Reserve University, Cleveland, OH USA

**Keywords:** Quality improvement, Implementation science, Evaluation, Delivery of healthcare

## Abstract

**Background:**

In healthcare change interventions, on-the-ground learning about the implementation process is often lost because of a primary focus on outcome improvements. This paper describes the Learning Evaluation, a methodological approach that blends quality improvement and implementation research methods to study healthcare innovations.

**Methods:**

Learning Evaluation is an approach to multi-organization assessment. Qualitative and quantitative data are collected to conduct real-time assessment of implementation processes while also assessing changes in context, facilitating quality improvement using run charts and audit and feedback, and generating transportable lessons. Five principles are the foundation of this approach: (1) gather data to describe changes made by healthcare organizations and how changes are implemented; (2) collect process and outcome data relevant to healthcare organizations and to the research team; (3) assess multi-level contextual factors that affect implementation, process, outcome, and transportability; (4) assist healthcare organizations in using data for continuous quality improvement; and (5) operationalize common measurement strategies to generate transportable results.

**Results:**

Learning Evaluation principles are applied across organizations by the following: (1) establishing a detailed understanding of the baseline implementation plan; (2) identifying target populations and tracking relevant process measures; (3) collecting and analyzing real-time quantitative and qualitative data on important contextual factors; (4) synthesizing data and emerging findings and sharing with stakeholders on an ongoing basis; and (5) harmonizing and fostering learning from process and outcome data. Application to a multi-site program focused on primary care and behavioral health integration shows the feasibility and utility of Learning Evaluation for generating real-time insights into evolving implementation processes.

**Conclusions:**

Learning Evaluation generates systematic and rigorous cross-organizational findings about implementing healthcare innovations while also enhancing organizational capacity and accelerating translation of findings by facilitating continuous learning within individual sites. Researchers evaluating change initiatives and healthcare organizations implementing improvement initiatives may benefit from a Learning Evaluation approach.

**Electronic supplementary material:**

The online version of this article (doi:10.1186/s13012-015-0219-z) contains supplementary material, which is available to authorized users.

## Introduction

In response to the landmark Institute of Medicine Report, *Crossing the Quality Chasm—A New Health System for the 21st Century* [1], there have been widespread attempts to transform the US healthcare system to achieve the triple aim of improved health, improved patient experience, and reduced cost of care [2]. Primary care practices across the US are engaged in improvement initiatives, including demonstration projects to implement patient-centered medical home principles [3,4] or new models of care delivery, such as integrating primary and behavioral healthcare [5-7]. Healthcare organizations, researchers, funders, and policy makers have a unique opportunity to learn from both interventions and natural experiments [8-12].

Learning from practice and system quality improvement efforts is challenging; too often they fail to achieve projected outcomes [13,14]. Quality improvement projects are rarely rigorously evaluated; therefore, reasons for not achieving anticipated results are unknown and much of the on-the-ground learning is lost [10,15-17]. Although demonstration projects are evaluated, making sense of outcomes can be challenging because of differing and changing contexts in which improvements are implemented [18,19]. In part, this is because traditional quantitative approaches used to evaluate healthcare innovations are guided by *a priori* specification of hypotheses, sampling, measures, and randomization; standards that are often hard to attain in the changing contexts of real-world settings. It is critically important to evaluate the iterative process of system change inherent in demonstration projects and to capture and apply lessons learned for further improvement. Thus, imposing traditional scientific principles and *a priori* specifications on evaluation design may constrain our ability to pay attention to context and transportability.

Flexible, scientifically rigorous study designs are needed to evaluate demonstration projects and large-scale quality improvement efforts. These designs must be rigorous in their ability to compare outcomes with good internal validity, in helping healthcare systems learn from implementing small cycles of change to enhance their own efforts and in continually assessing the implementation process and the context in which changes are made. These innovative study designs must help evaluate implementation and outcomes, while also enhancing the success of implementation [18,19] and accelerating translation of findings to other settings.

In this paper, we present Learning Evaluation as a methodological approach to address these needs. Learning Evaluation blends quality improvement and implementation research methods with an emphasis on drawing systematic and transportable lessons from healthcare innovations implemented across multiple organizations in changing, real-world settings. Two key aspects of this approach set it apart from other evaluation approaches; its emphasis on facilitating learning from small, rapid cycles of change within organizations and on capturing contextual and explanatory factors related to implementation and their effect on outcomes across organizations. We describe Learning Evaluation and illustrate its application to evaluating the Advancing Care Together (ACT) program [11,20] —a 3-year initiative of 11 practices implementing evidence-based interventions to integrate behavioral health and primary care.

### The Learning Evaluation approach

The overarching idea underlying Learning Evaluation is that assessment needs to be flexible, grounded, iterative, contextualized, and participatory in order to foster rapid and transportable knowledge. This approach integrates the implementation and evaluation of interventions by establishing feedback loops that allow the intervention to adapt to ongoing contextual changes. When separate healthcare organizations (e.g., primary care clinics, hospital units) participate in large-scale change initiatives through a research study or demonstration project, the Learning Evaluation approach is designed to balance the flexibility needed for within-system innovation and the structure needed to support rigorous evaluation, cross-organization learning, and generate transportable findings. Learning Evaluation is founded on five key principles. Table 1 summarizes these principles, their rationale, and ways to assess them.

**Table 1:** **Principles underlying the Learning Evaluation**

**Principles**	**Reason to assess principle**	**Ways to assess principle**
*Principle 1*: Gather data to describe types of changes made by healthcare organizations, how changes are implemented, and the evolution of the change process	To establish initial conditions for implementing innovations at each site and to describe implementation changes over time	- Interview with healthcare organizations to establish detailed understanding of the plan for implementing change at baseline by engaging organizational leaders
		- Use mixed methods to monitor how this plan evolves
*Principle 2*: Collect process and outcome data that are relevant to healthcare organizations and to the research team	To engage healthcare organizations in research and in continuous learning and quality improvement	- Identify target populations and process and outcome measures of interest to organizations
		- Identify relevant process measures to track for selected target populations
		- Track performance on selected measures at regular time intervals throughout implementation
*Principle 3*: Assess multi-level contextual factors that affect implementation, process, outcome, and transportability	Contextual factors influence quality improvement; need to evaluate conditions under which innovations may or may not result in anticipated outcomes	- Collect qualitative and quantitative contextual data in real time
		- Conduct rigorous analysis to identify key contextual factors affecting outcomes
*Principle 4*: Assist healthcare organizations in applying data to monitor the change process and make further improvements	To facilitate continuous quality improvement and to stimulate learning within and across organizations	- Synthesize, summarize, and share data with organizations at regular intervals
		- Discuss data with leaders to stimulate further improvement
		- Assist organizations in learning from their own data to refine their innovations with a focus on continuous learning
*Principle 5*: Operationalize common measurement and assessment strategies with the aim of generating transportable results.	To conduct internally valid cross-organization mixed methods analyses	- Harmonize process and outcome measures across organizations by engaging organizational leaders
		- Create a set of common measures relevant to all organizations (e.g., screening rates). This allows meaningful statistical and qualitative comparisons across organizations

**Figure 1:**
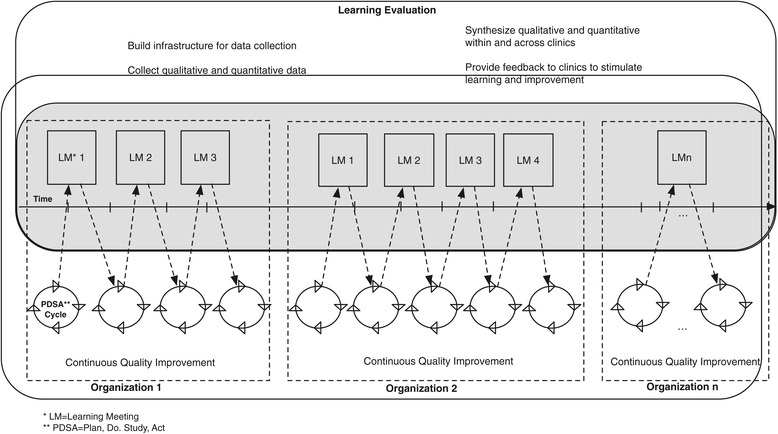
**Learning Evaluation.**

The design of a ‘typical’ Learning Evaluation for a demonstration project appears in Figure 1. In this depiction, we use the term ‘organization’ as an example of a healthcare organization participating in a change effort (e.g., primary care clinic, hospital intensive care unit, mental healthcare center). Each participating organization (*depicted in dashed rectangles*) implements several, short quality improvement Plan-Do-Study-Act (PDSA) cycles (*depicted by circles*) to make incremental change toward a common program goal (e.g., integrating primary care and mental healthcare, transforming to a patient-centered medical home). The number of PDSA cycles can vary from organization to organization.

Within each organization, the evaluation team collects qualitative and quantitative data to evaluate success of these PDSA cycles using established quality improvement research methods such as run charts and root cause analyses. Qualitative data collection is designed to assess the local, context-specific character of each organization (e.g., what is the context in which the change occurs, what factors stimulate subsequent change) and to identify the context-generalizable features of the change across organizations. Multiple organizations working toward the same improvement target and participating in a consistent qualitative assessment facilitates identification of transportable features, i.e., barriers, facilitators, and solutions identified through the change process. Quantitative data collection is designed to identify common process of care measures that are relevant to organizations such that each organization can use these data to set targets for their next quality improvement cycle. Also, change in the process of care measures over time demonstrates success in implementing strategies.

During the Learning Evaluation, qualitative data are collected and reported in real time while quantitative data, although collected continuously, are reported to the evaluation team at fixed intervals. The evaluation team processes the quantitative data and examines changes in processes of care measures over the previous interval. These findings are synthesized with qualitative data on implementation factors and shared with each organization at learning meetings (*depicted in rectangles as LM1, LM2, and LMn*). Organizational staff are encouraged to reflect on findings and plan next steps in the quality improvement (*depicted by dashed arrows*). Implementation lessons emerging from other organizations are shared to inform change efforts at another organization. The overarching work done by the evaluation team throughout the duration of the program (*depicted in rectangle at the top*) includes building infrastructure within and across organizations to facilitate qualitative and quantitative data collection, synthesizing these data, providing feedback, and conducting cross-case comparative analyses to generate transportable findings to inform future implementation.

### Application of Learning Evaluation

The Advancing Care Together (ACT) initiative provides an illustration of how the Learning Evaluation approach can be used. Below, we describe how we applied the five principles outlined in Table 1 to evaluate the ACT program. This includes collecting qualitative data to assess types of changes made by participating practices and how they implemented changes (principle 1); identifying clinically relevant process of care and outcome data to monitor change (principle 2); assisting practices in making sense of these data and using them in the improvement process (principle 3); assessing contextual factors affecting implementation at multiple levels (principle 4); and operationalizing common measures and our plans for a cross-project mixed methods analysis to generate transportable findings (principle 5).

### The ACT program

A large body of literature establishes the individual, population, and system level benefits of integrating behavioral health and primary care [5,21,22]; yet few practices have on-site behavioral health and primary care teams working together to deliver unified care to patients. The ACT program, funded by The Colorado Health Foundation, was designed to transform the delivery of healthcare by learning from 11 demonstration projects about what it takes to implement models of integrated care in real-world settings. Nine primary care and two mental health organizations were selected for 3-year projects to implement evidence-based strategies to integrate care. Sites were purposively selected to represent diversity with respect to practice type, size, business model, and patient panel served; thus maximizing our ability to draw transferrable insights about factors that facilitated and hindered integration. Each organization, with help of the evaluation team, selected one practice in which to implement their evidence-based integration strategies. The 11 practices included over 440 practice members and 120,000 patients. Practices varied on the evidence-based strategies they implemented; which included one or more of the following: systematic screening for primary care and/or behavioral health needs using evidence-based screening tools (e.g., Patient Health Questionnaire-9 [PHQ9], Generalized Anxiety Disorder-7 [GAD7], Body Mass Index [BMI]), a shared medical record for recording and sharing patient information, and co-locating primary care and behavioral care professionals in the same site. Five practices integrated behavioral healthcare into primary care while others focused on improving their existing integration efforts. Additional characteristics of participating practices and the innovations they implemented are provided in Additional file 1.

The ACT Learning Evaluation was carried out by a transdisciplinary team, with expertise in epidemiology, qualitative research methods, biostatistics, practice-based research, practice facilitation, healthcare policy, health economics, anthropology, and integrated care. The University of Texas Health Science Center at Houston and the Oregon Health & Science University Institutional Review Boards approved the study protocol.

### Types of data collected

Qualitative and quantitative data at multiple levels (e.g., practice, provider, and patient) were collected concurrently, analyzed in real time, and fed back to practices to inform ongoing change efforts. The evaluation team worked closely with practice leadership to tailor data collection to each site’s innovation and ensure data would benefit both individual practices and the evaluation team’s cross-project analysis. Table 2 provides a detailed description of types of data collected and their sources.

**Table 2:** **Types of data and methods of data collection employed in the Learning Evaluation of the Advancing Care Together (ACT) study**

**Primary data type**	**Description of data**	**Data collection process**
*Documents*	Documents collected included: call for proposals, notes from program office-sponsored meetings, and email communications when available; grant applications and grantee reports; as well as manuscripts, training materials, grantee presentations.	• We collected documents throughout the study period. The program office and grantees shared documents with us freely.
*Online diaries*	The online diaries produced written documentation of what was observed and experienced during implementation of each intervention. Several hundred pages of rich description of implementation processes was documented across practices using the online diaries.	• We worked with practice leaders to identify people who were closely involved with implementing integration strategies and who could write about their observations/experiences during implementation.
		• Four to six people were identified from each practice
		• Each practice had a private diary room. Only the diary keepers and the evaluators had access to the room.
		• Diary keepers posted entries approximately every 2 weeks. Posted entries were viewed and responded by other diary keepers from the team.
		• Evaluators also interacted with diary keepers in real-time, asking questions, and discussing and responding to diary entries.
		• Diary data was collected throughout the study period.
*Site visits*	Evaluation team members conducted 2-day visits to each practice. Fieldnotes were prepared by the evaluators on the site visit to document observations about the practice and the integration strategies being implemented.	Evaluators conducted interviews with key informants at each practice. Additionally, evaluators observed practice members doing the intervention at practices, when this was possible.
*Interviews*	Group interviews were conducted with practice members at program office-sponsored meetings. Fieldnotes were prepared to capture what was said during these interviews.	Interviews were conducted once a year (at baseline, 1 year into the study period, and at the conclusion of the study). When we had unanswered questions about an intervention, we scheduled a phone interview.
*Surveys*	A web-based survey was collected from each participating site to assess practice structure and function, including patient panel characteristics (socio-demographic and insurance), practice type and ownership, provider types, use of registries and clinical decision support systems, and existing practices pertaining to delivering integrated care.	Practice survey data was collected at baseline (pre-intervention). One person in the practice (e.g., Office Manager, lead physician) completed this survey.
*EHR data*	• Process of care measures (screening for behavioral and/or primary care conditions and receipt or referral for further counseling as needed) for target patients were collected to examine if implementing interventions resulted in improvements in care processes	Process measures were extracted from the EHR by a designated practice member and reported to the evaluation team every 3 months over a 1-year period.
	• Outcome measures were collected for screen-positive patients to examine if interventions resulted in change in outcomes	A designated practice member extracted visit-level data on outcome measures, socio-demographics, and comorbidity data for each patient who screened positive for primary and/or behavioral health condition at baseline and up to 6 months after end of evaluation period.

#### Evaluation pre-work

Prior to implementation, the evaluation team met with practice leaders to depict the process by which sites envisioned implementing their innovation. Additional file 2 provides an example of such an intervention process diagram. These diagrams helped identify key elements of interventions at baseline, provided a framework to guide measurement (i.e., what elements might create what types of changes that would need to be measured), helped evaluation team identify practice members with critical roles on-the-ground to participate in the online diary, and provided a starting point for following each intervention’s evolution. Intervention diagrams of all 11 practices were then reviewed by the evaluation team to glean similarities and differences in integration strategies and in the process of implementing them. Evaluators posed questions and sought clarifications from practice teams to crystallize the implementation process diagram. This helped to identify a common set of process of care and outcome measures to evaluate implementation and effectiveness within and across practices.

Based on these discussions, the evaluation team identified two processes of care measures common across all practices. These were screening for behavioral and/or primary care condition using evidence-based screening tools (e.g., PHQ9, GAD7, BMI) and referral for or receipt of further counseling for patients who screened positive. Additional file 3 lists primary care and behavioral health process of care measures collected by ACT practices. Practice intervention diagrams were used to create patient tracking sheets tailored to each site’s integration strategy. Additional file 4 provides an example of such a tracking sheet. Tracking sheets provided a data structure for practices to either create EHR queries or manually collect patient-level data on screening and receipt of further services/counseling in the practice or referral to other external services.

#### Collect qualitative data to establish initial conditions and describe change process (principle 1)

We collected qualitative data to answer four research questions: (1) What were the initial conditions to implement change among ACT practices? (2) What types of changes were they making to integrate care? (3) How were they making these changes, and what facilitated or hindered this process; and (4) What were key stakeholders’ (clinicians, clinical team members, patients) experiences with these changes? To answer these questions, we collected key documents, conducted observation visits, and interviewed stakeholders. We also asked practice members to participate in an online diary [23] where they posted entries about their implementation experience approximately every 2 weeks.

#### Collect quantitative data to assess process and outcome level changes (principle 2)

Quantitative data were collected to (1) obtain descriptive data on practice structural and functional characteristics, including patient panel characteristics, (2) estimate reach [9,24] of the implementation strategies, and (3) assess process and outcome measures to evaluate implementation success. We collected practice characteristics data through a survey completed by the office manager. Data on reach of the implemented strategies was gathered using a Reach Reporter (see Additional file 5) completed by practice members and returned to the evaluation team every 3 months. The Reach Reporter included data on the (1) number of target patients seen at the practice during the 3-month period, (2) number of target patients screened for targeted primary care or behavioral healthcare conditions, (3) number of screened patients who screened positive, and (4) number of patients screened positive who were referred for further care.

Process measures (e.g., practice-level rates of screening for behavioral or physical health condition, referral/counseling for additional services among screen-positive patients) were collected by practice members from the EHR or through manual tracking and reported to the evaluation team quarterly. Practices extracted and provided data on patient-level outcome measures (e.g., PHQ9, GAD7, BMI scores) from their EHR system at the end of the study period. Patient-level outcome measures differed between practices because of differences in target populations and integration strategies used. Therefore, a composite outcome that measured change in targeted outcomes across practice was calculated. Patient outcome measures are ascertained at baseline (start of the first quality improvement cycle) and at 6-month follow-up. Additional follow-up data points, if available, are also used to examine long-term changes.

#### Assess multi-level contextual and explanatory factors (principle 3)

The evaluation team processed qualitative and quantitative data concurrent with collection to understand the implementation experience.

### Tracking and analyzing implementation events

The evaluation team received diary data in real time as practice members made entries about the changes they were making. Evaluators received an email notification for each new diary post. The evaluation team met weekly to discuss diary posts and progress at each site. This allowed us to make sense of the intervention experience as it was unfolding, to identify questions and respond to posts seeking answers to those questions, and to triangulate with other data sources, such as documents and site visit data, to get a coherent picture of the intervention and implementation experience at each practice. This prospective preliminary analysis of qualitative data helped us update intervention figure and tracking sheets for each site in real time. This helped us reflect on intervention changes and to determine when to conduct a site visit. Additionally, we monitored who was and was not posting on the diaries and sought out other ways to engage those who were silent on the diaries (e.g., emailing people outside the diary, holding informal telephone conversations). These conversations were written up as field notes or recorded and transcribed; they were included in our database and discussed at weekly team meetings.

### Creating run charts and mapping implementation events

Synchronously with process evaluation activities, practices completed a series of rapid PDSA quality improvement cycles [15] to implement their proposed interventions. Every 3 months, practices reported rates of screening and receipt/referral for counseling. The evaluation team cleaned, processed, and plotted screening and referral/counseling rates on a time series to create run charts [25] for each practice. Use of run charts along with identification of causes (i.e., implementation events) of variation is a standard quality improvement research method to evaluate change [25,26]. For each 3-month period, we used concurrently collected qualitative data to identify and map key implementation events (e.g., change in workflow, change in screening strategy, behavioral professional turnover) that could explain variation in run chart measures.

#### Audit and feedback to help practices reflect and stimulate further change (principle 4)

Each individual site’s performance on screening and referral/counseling rates was shared with practice leaders during quarterly 1-h learning meetings with the evaluation team. Qualitative and quantitative data were presented in an easy to understand report that was shared and reviewed with practice members in advance of the learning meeting (see Additional file 6). Practice members reflected on their on-the-ground experiences integrating care in the context of their reported screening and referral rates. They discussed the strategies that worked and did not work and as needed, identified changes to test in PDSA cycles in the next 3-month period. This iterative process repeated quarterly until the end of the funding period; some practices continued even after end of funding.

#### Use mixed methods to generate valid and transportable findings (principle 5)

Our mixed methods analysis was informed by the Realist approach and the work of Pawson and Tilley [27]. Rather than the traditional approach of evaluating changes in outcome produced by interventions across the group of practices, we instead focused on settings (i.e., context) that were successful and unsuccessful at implementing integrated care and on identifying the mechanisms that led to successful implementation and changed intermediate outcomes. Within the context of a multi-site demonstration project conducted in real-world settings, it was not feasible to randomize sites or to specify target patient samples or measures *a priori*. Therefore, we incorporated several design and analysis elements to enhance scientific rigor, including rigor in study design and analysis.

##### Rigor in study design

Since each practice implemented an intervention, the study design within each participating site was a single group pre-post quasi-experimental study. History (events occurring concurrently with intervention) and maturation (naturally occurring changes over time that could explain treatment effect) are the two most common threats to internal validity in quasi-experimental studies [28]. To counteract these threats, the Learning Evaluation incorporates several design elements. First, we included measurement of quantitative outcomes at multiple time points over the course of the study along with detailed collection of qualitative data on implementation events. This allowed us to examine if observed variations in process outcomes (screening and referral) were related to changes made in practices and not due to other concurrently occurring events [28]. Further, the design incorporated ‘member checking’ when the evaluation team shared feedback reports with practice leadership during quarterly learning meetings. Practice members validated or discredited our data, providing additional clarity to our findings. This process was facilitated by developing rapport with practice leaders early on in the study and demonstrating how the evaluation data could help them refine their innovations.

##### Rigor in analysis

We analyzed qualitative and quantitative data with the aim of integrating findings, rather than analyzing each source of data separately. Triangulation of data sources is critical to rigor in mixed methods analysis. To do this, the evaluation team conducted cross-site comparative analyses using both types of data [9]. Qualitative data were entered in *Atlas.ti*™ and coded. Analysis proceeds through several iterations, first reviewing all text tagged within the same code, identifying prevalent themes first within the same code and then across codes. In a similar way, data analysis proceeds first within and then across practices to identify factors that hindered or facilitated implementation while also paying attention to the role contextual influences played, as is common in a realist evaluation approach [29]. Quantitative data on EHR-derived process and outcomes of care measures were first analyzed for patients within individual practices to evaluate if their ACT intervention led to significant improvements in physical and behavioral health outcomes. To do this, we constructed longitudinal growth models to examine pre-post change in outcomes, while exploring the effects of key confounding variables [30]. Next, we pooled EHR-derived patient-level data across practices and operationalize a standardized outcome measure for each patient (e.g., composite score for physical and behavioral healthcare service delivery). We then examined if implementing integrated care results in statistically significant change in the composite score across practices. Rather than simply examining changes in outcomes, we used a realist evaluation analysis approach to identify the specific integration approaches (mechanism) that explain observed outcomes (outcome) in the presence or absence of contextual factors (context) by conducting in-depth cross-site comparative analyses. To do this, qualitative and quantitative findings were synthesized together to identify and describe types of integration models associated with improved outcomes and contextual factors that facilitate or hinder implementation [9,16,31]. Although data analyses are still underway, in ACT, we hypothesized that practices that screened patients systematically had embedded behavioral health clinicians on the primary care team, had a path identified to refer patients for other specialty services, and had a shared mental model for integration and are more likely to have improved patient outcomes. Further, this relationship might differ by practice type (federally qualified health center, independent primary care practice, or integrated health system) such as hybrid federally qualified health center and community mental health center practices that may have additional resources to connect patients to needed care than independent primary care practices. Additional file 7 presents preliminary results from ACT using the methodology described above to identify types of integration approaches observed among practices. When outcome data are available, this method will help identify the mechanisms that result in improved outcomes and the contextual factors that modify the observed relationship.

Together, these steps describe how we applied the five principles of the Learning Evaluation to ACT. The Learning Evaluation approach results in facilitating and expediting change within practices, draws cross-cutting findings from all participating sites, identifies effective models of integrated care that can be successfully implemented, and identifies multi-level contextual factors to pay attention to future dissemination of effective models.

## Discussion

Learning Evaluation is a rigorous approach that blends evaluation theories and research methods to comprehensively assess implementation of healthcare innovations across organizations. Evaluating complex interventions implemented across multiple sites (as in demonstration projects) creates methodological challenges to generate overarching findings that have both internal and external validity [19]. New scientifically rigorous methods are needed to evaluate such projects.

Scientific rigor in the Learning Evaluation approach comes from conducting *within*-*organization* evaluations of changes in process and outcomes measures coupled with a mixed-methods evaluation of *between*-*organization* change. *A priori* specification of eligible populations and outcomes across sites is one way to achieve this objective. However, this may not be feasible or desirable in real-world situations where healthcare organizations benefit from selecting target populations and outcomes that are most relevant to their context. For instance, in ACT, many practices selected screening with PHQ9 as the outcome of interest; however, a few also selected BMI or HbA1c. Regardless of the outcome, we measured rates of screening combining PHQ9, BMI, and other methods of identifying patient need, as a measure of implementation success. Ongoing learning and adaptation of measurement allows both rigor and relevance.

Another source of rigor in this design is the purposeful measurement of quantitative process and outcomes at multiple time points during implementation along with concurrent but independently collected qualitative data. Together, these data provide estimates of change in outcomes, elements of the intervention, contexts, and other explanatory factors that impact implementation. The use of qualitative and quantitative data mitigates the effects of history and maturation. Addition of a comparison group of practices could also enhance scientific rigor by allowing assessment of broader secular changes. Finally, conducting rigorous comparative case studies by analyzing data iteratively within a case and then across cases helps identify contextual factors and mechanisms associated with observed outcomes.

The Learning Evaluation blends established quality improvement and implementation research methods. In ACT, individual practices implemented short PDSA quality improvement cycles to refine integrated care delivery. The evaluation team used run charts and qualitative data to help practices reflect on what worked or did not to inform the next PDSA cycle [25,26]. Data were analyzed in real time to feed back to innovators. While individual practices completed their own quality improvement cycles, evaluators identified similarities and differences in implementation approaches and barriers and facilitators of those approaches across organizations. This process of facilitating quality improvement at participating practices while generating cross-site implementation findings can help accelerate the research translation pipeline. It overcomes one challenge of traditional research designs by ensuring that we do not wait until the end of a multi-year change initiative to disseminate and apply the lessons learned [11]. On-the-ground strategies such as the Learning Evaluation can help healthcare systems and practices enhance their change efforts in real time [32-34] and contribute to transportable implementation lessons.

The Learning Evaluation draws on two established evaluation theories: empowerment evaluation, [35,36] which focuses on helping innovators improve their change initiatives by encouraging self-evaluation and reflection; and realist evaluation [27,37], which focuses on bringing a better understanding of the context and the mechanisms that generate outcomes. Learning Evaluation shares features with empowerment evaluation in that the evaluation team works collaboratively with innovators to develop data collection strategies and routine processes for jointly sharing and reflecting on data to foster continuous learning, improvement, and advocacy for policy changes. For ACT practices, the Learning Evaluation method informed current quality improvement activities and developed capacity for data collection and monitoring for future efforts. This process helped evaluators identify cross-organization implementation findings and created a co-learning process. The realist evaluation approach emphasizes the importance of attending to the context in which interventions are implemented [27,37]. Thus, it is not only important to know ‘does it work?’ but also ‘what works in which conditions for whom?’ Principle 3 of our Learning Evaluation focuses on assessing relevant contextual factors at multiple levels; principle 5 looks across settings to analyze the influence of these factors on implementation. Data on contextual factors were collected at the start of the ACT program through evaluation pre-work, continued in real time while interacting with innovators through diaries and interviews and in learning meetings. This process and nature of the interaction between the evaluation team and practices is a novel aspect of the Learning Evaluation approach. Triangulating qualitative implementation data and quantitative process and outcome data in real time, at regular intervals, and across organizations is another innovative aspect of this design. Finally, this approach provides rich, in-depth information on the context in which change occurs, which can then be incorporated into mixed-methods analyses [18] to help others understand how they might transport or knowledgeably reinvent an intervention approach in a different context.

Although both empowerment and realist evaluation approaches are applicable to our design, it is their blending that is a unique aspect of Learning Evaluation. As illustrated in the ACT evaluation, this blending helped evaluators empower innovators with cross-organization knowledge that could inform each site’s future PDSA cycles. At the same time, evaluators paid close attention to contextual factors within and across organizations so that effective models of care can be translated with an in-depth knowledge of the conditions in which they work. Other evaluation theories, such as Ovretviet’s action evaluation approach [38], emphasize this principle of collecting and providing data to organizations to help them decide on further action. However, it is not designed to generate transportable findings. Batalden’s Serial ‘V’ approach [39] also shares similarities with Learning Evaluation in its integration of continual improvement, process improvement, and outcome measurement. However, neither of these approaches was developed for evaluating implementation across multiple organizations or generating transportable findings.

A few specific considerations related to the use of Learning Evaluation are worth noting. First, some healthcare organizations may not be able to collect the data initially agreed upon. However, the flexibility in Learning Evaluation allows evaluators to assist organizations by suggesting alternative ways gleaned from other participating sites to collect data, thus enhancing their data collection capacity for future initiatives. Second, the emphasis on engagement and openness to learning is crucial for its success. Thus, it is important for healthcare organizations participating in such an evaluation to be committed to it. In ACT, facilitating the evaluation objectives was a prerequisite for funding innovators. Finally, the Learning Evaluation approach may feel to some to be at odds with current standards of rigor, which value fidelity to *a priori* hypotheses and methods. These are at odds with methods that emphasize ongoing learning and paying attention to context as crucial to understanding observed outcomes. However, as illustrated in the ACT example, Learning Evaluation is actually complementary to current approaches and adds contextualized, ongoing knowledge that is essential to rapidly advancing implementation science.

The Learning Evaluation is not a ‘canned’ approach to evaluating healthcare innovations, but it involves the flexible application of five general principles (Figure 1, Table 1) designed as a highly adaptable approach to rapid, relevant, rigorous evaluation and shared learning that is at the heart of the IOM’s learning system concept [40,41]. Others who may want to replicate this approach can use the general principles and adapt them to their specific contexts. Adhering to the principles can help a single system or a multi-site demonstration project collect data and report findings that are highly transportable and that also provide contextual understanding for others who wish to reinvent their interventions.

Learning Evaluation is relevant not only for researchers/evaluators but also for healthcare systems, policy makers, and funders. A key consideration for researchers is that data are shared with innovators regularly and often. This requires researchers to be flexible and nimble in adapting their approach when proposed innovations are modified to fit the local context. An important focus of Learning Evaluation is to increase the capacity of healthcare systems to make and sustain change, for example, by helping develop data tracking and monitoring capacities. This level of engagement of evaluators with innovators is a unique aspect of this approach [40-42]. Learning Evaluation aligns with the quality improvement process and provides a clinically relevant and realistic way to evaluate health system changes. This approach is especially relevant for policy makers; it is ideally suited for evaluating demonstrations of new policy initiatives (e.g., tests of payment models) in which it is critical to assess context in which changes occur so that dissemination can be targeted to healthcare systems positioned to deliver new models of care. Finally, Learning Evaluation extends methodological knowledge and provides a scientifically rigorous framework to evaluate healthcare innovations funded by local health systems as well as national agencies such as the Agency for Healthcare Research and Quality (AHRQ) [43].

## Conclusion

Learning Evaluation can be used to facilitate continuous learning, generate systematic and rigorous findings about implementing healthcare innovations, and add contextualized, ongoing knowledge which is essential to rapidly advancing implementation science.

## Additional files

Additional file 1:
**ACT practice and innovation characteristics.**
Additional file 2:
**Intervention process diagram.**
Additional file 3:
**Process and outcome measures.**
Additional file 4:
**Patient tracking sheet.**
Additional file 5:
**Reach Reporter.**
Additional file 6:
**Feedback report to ACT practices.**
Additional file 7:
**ACT intervention typology.**
