# Supplementary material for: Learning Evaluation: blending quality improvement and implementation research methods to study healthcare innovations
Source: Implement Sci. 2015 Mar 10;10:31. doi: 10.1186/s13012-015-0219-z (PMC4357215; doi:10.1186/s13012-015-0219-z)
Supplement: Additional file 1: — ACT practice and innovation characteristics. [file 13012_2015_219_MOESM1_ESM.pdf]

Appendix A: ACT innovator clinic and intervention characteristics

Table 1: Characteristics of ACT clinics

**Clinic Characteristics**	**N=11**
**Clinic type**	
**Primary care clinics**	9
Solo	1
Single specialty	4
Multispecialty	5
**Mental health clinics**	2
**Ownership**	
Federally qualified health Center	4
Community Mental Health Center	2
Clinician	4
Health maintenance organization	1
**Location**	
Rural	3
Urban	8

Table 2. Description of ACT Innovations

ID	Description of ACT Innovation
1	A post-doctoral psychology training program built on their existing partnership with an FQHC that provides health care services to seniors. They refined and implemented an enhanced, computerized cognitive and psychological screening as part of routine patient care. The team developed a summary report of the screening (e.g., like a lab report), refined the process for routine review by the medical team, and tracked clinical use of the screening by creating an EHR tracking interface. Leadership of the post-doctoral training program attended primary care clinic meetings to encourage bi-directional communication between medical and behavioral health providers.
2	This privately owned primary care practice expanded current behavioral health screening processes by implementing electronic tablets during patient check-in. Practice leadership worked with information technology partners to develop coding necessary to integrate screening data directly into their EHR. Concurrent with ACT, the practice was able to leverage resources from other change initiatives and hire two co-located behavioral health providers.
3	A CMHC developed a new integrated care clinic; this included building a new facility and bringing on a primary care team. The used systematic screening for behavioral health and medical need at the time of patient check in using an electronic screening tablet. The CMHC developed a Personal Health Profile that extracts selected information from the CMHC's two separate behavioral health and medical EHRs to make information about patients' behavioral and physical health needs accessible at the point of care.
4	A private behavioral health center expanded an existing relationship with a private family medicine clinic by embedding behavioral health clinicians into the primary care clinic. They implemented routine screening for patients coming for annual, hypertensive, or diabetes care visits. The intervention included brief consultations with behavioral health providers in primary care and/or referral for more intensive care to a private behavioral health partner organization located in the same building.
5	A research team worked with their FQHC to implement a patient-reported tool that asks them to select one health goal (including health behavior risk reduction, medication management, and managing pain and stress/anxiety) they would like to work on. Clinic and research staff implemented the tool in a practice with an existing onsite behavioral health provider and newly added patient navigators. The project goal was to establish routine assessment of patients' health goals and referral as needed.
6	This privately owned primary care practice implemented routine screening for primary care (HbA1c among diabetics and BMI) and behavioral health (depression, anxiety, and substance use) issues using evidence-based screening tools. They partnered with a CMHC to hire, train, and supervise a co-located behavioral health provider and also included health coaching services to counsel at-risk patients.
7	A CMHC partnered with an FQHC and a substance abuse treatment center to deliver primary care, mental health, and substance use services. Specifically, they created a team of primary care professionals (i.e., physician's assistant, medical assistant, a care coordinator and a substance abuse counselor) and embedded it in the CMHC clinic.
8	This health system expanded their approach to integrate care by co-locating behavioral health providers in primary care clinics. They screened patients for depression using PHQ9 at discretion of primary care providers. The integrated behavioral health providers provided patients with solution-focused interventions and facilitated referrals to other organizational resources (e.g., long term therapy, case management). These behavioral health providers also provided some consultations to primary care clinicians upon request.
9	This solo, privately owned primary care practice embedded a behavioral health provider into the practice, employed systematic screening, and developed referral protocols.
10	This FQHC focused on increasing screening and behavioral health services for newly identified pregnant women by adding a psychology fellow. The fellow, with the support of other behavioral health providers at the practice, screened patients, offered brief therapy, and followed-up with eligible patients as needed.
11	A CMHC added a full-time substance abuse counselor to a FQHC where they already had an embedded behavioral health clinician. Organizational challenges resulted in the CMHC removing embedded services from the FQHC and developing primary care capacity in one of their behavioral health clinic service locations.

Abbreviations: CMHC = Community Mental Health Center; FQHC = Federally Qualified Health Center; SBIRT = Screening, Brief Intervention, and Referral to Treatment; EHR = Electronic Health Record; Comm = Communication; Coord = Coordination; Collab = Collaboration.
