# Supplementary figures and images for: Learning Evaluation: blending quality improvement and implementation research methods to study healthcare innovations
Source: Implement Sci. 2015 Mar 10;10:31. doi: 10.1186/s13012-015-0219-z (PMC4357215; doi:10.1186/s13012-015-0219-z)

Appendix C - Intervention Process Diagram Example

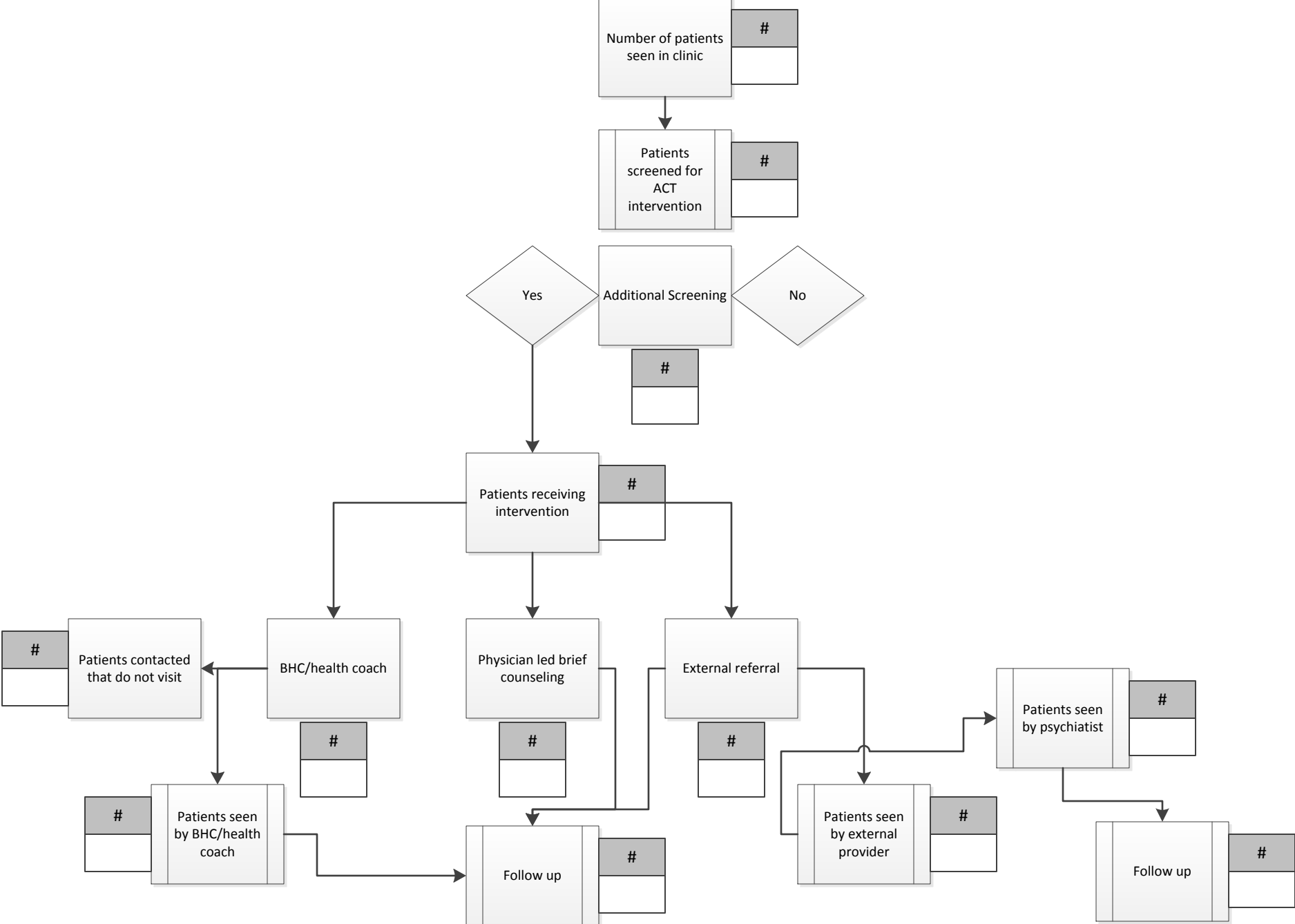

Supplement: Additional file 2: — Intervention process diagram. [file 13012_2015_219_MOESM2_ESM.pdf]
