# Supplementary material for: Learning Evaluation: blending quality improvement and implementation research methods to study healthcare innovations
Source: Implement Sci. 2015 Mar 10;10:31. doi: 10.1186/s13012-015-0219-z (PMC4357215; doi:10.1186/s13012-015-0219-z)
Supplement: Additional file 3: — Process and outcome measures. [file 13012_2015_219_MOESM3_ESM.pdf]

Appendix D: Process and outcome measures collected by ACT clinics

Site ID	Process of care measures (Screening for behavioral and/or medical condition)	Patient outcome measures
1	Cognitive screens	Not measured
2	Depression	PHQ9 scores
3	depression, anxiety, risky alcohol and substance use	PHQ9, GAD7, AUDIT, DAST scores
4	Depression	PHQ9 scores
5	unhealthy health behaviors	Not measured
6	Depression, alcohol use, obesity, diabetes	PHQ9, AUDIT scores, reductions in BMI and HbA1c
7	alcohol and substance use	DAST, AUDIT
8	depression	PHQ9
9	Depression, Alcohol and tobacco use, safe at home	PHQ9, AUDIT scores, smoking cessation
10	Depression, anxiety, alcohol and tobacco use, diabetes	PHQ9 scores
11	Substance use	DAST, AUDIT
