# Supplementary material for: Learning Evaluation: blending quality improvement and implementation research methods to study healthcare innovations
Source: Implement Sci. 2015 Mar 10;10:31. doi: 10.1186/s13012-015-0219-z (PMC4357215; doi:10.1186/s13012-015-0219-z)
Supplement: Additional file 4: — Patient tracking sheet. [file 13012_2015_219_MOESM4_ESM.pdf]

Appendix E: Example of a Patient Tracking Sheet

Dummy ID	Medical Record Number	Date of Visit	Date of Screening	Screen positive		Referral?				Intensive counseling?				Follow up?
**Please use the correct dummy ID for patients who screened positive**	**! This column is for your records only. Please maintain a copy of this file with this number** Please do not send files with medical record numbers or patient names.	Please provide the date of this visit	Please provide date patient was most recently screened **on or prior** to date of visit (Column C)	What behavioral test did patient screen positive for? 1=PHQ9>4 2=AUDIT>8 3=Both 4=don't know	What medical test did patient screen positive for? 1=**BMI≥30** 2=HbA1c>7 3=Both 4=don't know	If this patient's screen was "positive" did the patient receive a **traditional referral**? 1=Yes 2=No 3=Don't know	If this patient's screen was "positive" did the patient receive a **referral with outreach**? 1=Yes 2=No 3=Don't know	If this patient's screen was "positive" did the patient receive a **warm handoff referral?** 1=Yes 2=No 3=Don't know	If this patient's screen was "positive" did the patient receive a **staff or self-referral?** 1=Yes 2=No 3=Don't know	If referred, did patient receive intensive counseling from **primary care clinician**? 1=Yes 2=No 3= Don't know	If referred, did patient receive intensive counseling from **psychiatrist**? 1=Yes 2=No 3=Don't know	If referred, did patient receive intensive counseling from **health coach?** 1=Yes 2=No 3=Don't know	If referred, did patient receive intensive counseling from **behavioral health counselor?** 1=Yes 2=No 3=Don't know	If referred, was patient followed-up? 1=Feedback from psychiatry 2=Feedback from HC/BHC 3=No show 4=Don't know
		9/5/2013	8/28/2013	1	4	2	2	2	1	3	2	2	2	4
		9/19/2013	8/21/2013	1	4	2	2	2	1	3	2	2	1	2
		9/26/2013	9/20/2013	4	4	2	2	2	1	3	2	2	1	2
		9/26/2013	9/24/2013	1	4	2	2	2	1	3	2	2	1	2
		10/24/2013	10/15/2013	4	4	2	2	2	1	3	2	2	1	2
		10/24/2013	10/7/2013	1	4	2	2	2	1	1	2	2	1	2
		11/8/2013	10/17/2013	1	4	2	2	2	1	1	2	2	1	2
		11/11/2013	10/30/2013	4	4	2	2	2	1	3	3	2	1	2
		11/11/2013	11/18/2013	4	4	2	2	2	1	1	2	2	1	2
		11/12/2013	11/4/2013	4	4	2	2	2	2	3	2	1	1	2
