# Supplementary material for: Learning Evaluation: blending quality improvement and implementation research methods to study healthcare innovations
Source: Implement Sci. 2015 Mar 10;10:31. doi: 10.1186/s13012-015-0219-z (PMC4357215; doi:10.1186/s13012-015-0219-z)
Supplement: Additional file 5: — Reach Reporter. [file 13012_2015_219_MOESM5_ESM.pdf]

Appendix B – Example of an ACT Reach Reporter

START HERE

Step 1

What is the total *__number of active__* *patients in your practice for the period from* ***__11/15/2011 through 11/14/2012__*** *?*

Step 2

What *__percent__* *of your active patients (from Step 1) are...*

Enter values as percents ***without*** the percent symbol. (e.g., 99.0 for 99.0%).

Male	Female	Age 0-17	18-20	21-44	45-64	65+	Private insurance	Medicare	Medicaid	Uninsured	Other	African American or Black	American Indian or Alaska Native	Asian	Hispanic or Latino	White	Other	Unknown race/ ethnicity
1.01	*98.99*																	

Step 3 *PLEASE CONTACT THE EVALUATION TEAM TO AFFIRM OR CORRECT THE TARGET POPULATION DESCRIPTION BELOW.*

Briefly describe the *__target population__* *for your ACT innovation.*

All adult patients seen in your practice (18 years and over)

Step 4 *PLEASE CONTACT THE EVALUATION TEAM TO AFFIRM OR CORRECT THE SCREENING STRATEGY DESCRIPTION BELOW.*

List or briefly describe the *__screening strategies__* *(e.g., PHQ-9, GAD-7, AUDIT, chronic illness diagnosis) used with the above target population as part of your ACT innovation.*

PHQ9, AUDIT, BMI, HbA1c - These were selected based on the risk stratification table provided by you to the evaluation team

Go to **Step 5** on next tab, "B. ACT Reach Counts"

NOTE: Data entered on this worksheet are based on patients seen during your sampling reporting period. ***Your sampling period is 11/15/2012 through 02/15/2013 (Q1).***

Step 5

During the sampling period, what was the total number of all patients (unduplicated) seen in your practice?

Enter the actual number of patients seen.

Step 6

During the sampling period, how many target population patients were seen in the practice as part of your ACT innovation?

Enter actual numbers of patients (see sample values for male and female).

Male	Female	Age 0-17	18-20	21-44	45-64	65+	Private insurance	Medicare	Medicaid	Uninsured	Other	African American or Black	American Indian or Alaska Native	Asian	Hispanic or Latino	White	Other	Unknown race/ ethnicity	TOTAL (unduplicated)
999	9,999																		

Step 7

*Of the patients in **Step 6**, how many were screened (or otherwise evaluated) using any of the strategies listed in Step 4 from your target population as part of your ACT innovation?*

Enter actual numbers of patients.

Male	Female	Age 0-17	18-20	21-44	45-64	65+	Private insurance	Medicare	Medicaid	Uninsured	Other	African American or Black	American Indian or Alaska Native	Asian	Hispanic or Latino	White	Other	Unknown race/ ethnicity	TOTAL (unduplicated)

Step 8

*Of the patients in **Step 7**, how many screened positive (or otherwise were deemed eligible) to receive intervention services or strategies (e.g., counseling, referral, etc.) as part of your ACT innovation?* ***Your project defined screened positive as a patient who met one of the following criteria: PHQ9>4, AUDIT>8, BMI≥25, and HbA1c > 7, at least one chronic disease)***

Enter actual numbers of patients.

Male	Female	Age 0-17	18-20	21-44	45-64	65+	Private insurance	Medicare	Medicaid	Uninsured	Other	African American or Black	American Indian or Alaska Native	Asian	Hispanic or Latino	White	Other	Unknown race/ ethnicity	TOTAL (unduplicated)

Step 9

*Of the patients in **Step 8** who screened positive (or otherwise were deemed eligible), how many received intervention services or strategies (e.g., counseling, referral, etc.)?*

Enter actual numbers of patients.

Male	Female	Age 0-17	18-20	21-44	45-64	65+	Private insurance	Medicare	Medicaid	Uninsured	Other	African American or Black	American Indian or Alaska Native	Asian	Hispanic or Latino	White	Other	Unknown race/ ethnicity	TOTAL (unduplicated)
