# Supplementary material for: Learning Evaluation: blending quality improvement and implementation research methods to study healthcare innovations
Source: Implement Sci. 2015 Mar 10;10:31. doi: 10.1186/s13012-015-0219-z (PMC4357215; doi:10.1186/s13012-015-0219-z)
Supplement: Additional file 6: — Feedback report to ACT practices. [file 13012_2015_219_MOESM6_ESM.pdf]

Appendix F – Feedback Report to ACT Clinic
ACT Clinic
Reach and care process measures for ACT innovation
Sampling period – Nov 15, 2012 to Nov 15, 2013

ACT Clinic's innovation is focused on patients 18 years of age or older visiting the clinic during the study period. They aim to systematically screen target patients using PHQ2 followed by PHQ9 if PHQ2 positive (depression), AUDIT (substance use), BMI (obesity), and HbA1c (diabetes). Patients were assessed as screen positive if PHQ9>4, AUDIT>8, BMI≥30± and HbA1c>7.

The table below provides detailed screening, referral, and follow-up rates for ACT Clinic's innovation for the 12 month period from Nov 15, 2012 to Nov 15, 2013.

	Nov 15, 2012 to Feb 15, 2013	Feb 16 to May 15 2013	May 16 to Aug 15 2013	Aug 16 to Nov 15 2013
**Number of target patients**	**2,749**	**2,087**	**2,314**	**1,868**
**Screened, n (%)**	**2,407 (87)**	**1,746 (84)**	**1,779 (77)**	**1,609 (86)**
**Screened positive, n (%)**	1,775 (73.7)±	1,135 (65.0)±	856 (48.1)±	689 (42.8)
**Total number of target patients screened positive**	**1,775**	**1,135**	**856**	**689**
Referral*, n (%)	**72 (4.0)**	**78 (6.9)**	**147 (17.2)**	**176 (25.5)****
Traditional Referral to outside BH	5 (6.2)	2 (2.6)	58 (39.4)	0
Referral with outreach	68 (83.9)	16 (20.5)	83 (56.5)	167 (44.8)
Warm handoff	54 (66.7)	20 (25.6)	15 (10.2)	0
Self/Staff-Referral	20 (61.7)	48 (61.5)	44 (29.9)	9 (2.4)
Counseling for pts referred*, n (%)	**78 (54.9)**	**64 (82.0)**	**134 (91.1)**	**23 (13.1)**
Primary care clinician	33 (40.7)	9 (11.5)	104 (70.7)	3 (13.0)
Psychiatrist	6 (7.4)	0 (0)	0	0
Health coach**	26 (32.1)	43 (55.1)	36 (24.5)	15 (65.2)
Behavioral health counselor**	62 (76.5)	55 (70.5)	38 (25.8)	8 (34.8)
Counseling among all screen positive pts*, n (%)				**111 (16.1)**
Primary care clinician	33 (1.8)	18 (1.6)	719 (84.0)	88 (12.8)
Psychiatrist	6 (0.3)	0	0	0
Health coach**	26 (1.4)	116 (10.0)	74 (8.6)	16 (2.3)
Behavioral health counselor**	62 (3.4)	55 (4.7)	38 (4.4)	11 (1.6)
Follow-up among referred pts, n (%)	**73 (93.6)**	**55 (85.9)**	**71 (48.3)**	**23 (13.1)**

*patients may have gotten more than one type of referral or counseling, therefore rates may not add to 100%
**percentage out of three referral types—outreach, warm-handoff, and self/staff referrals
± screen positive definition for BMI changed from BMI≥25 to BMI≥30 after end of Q1

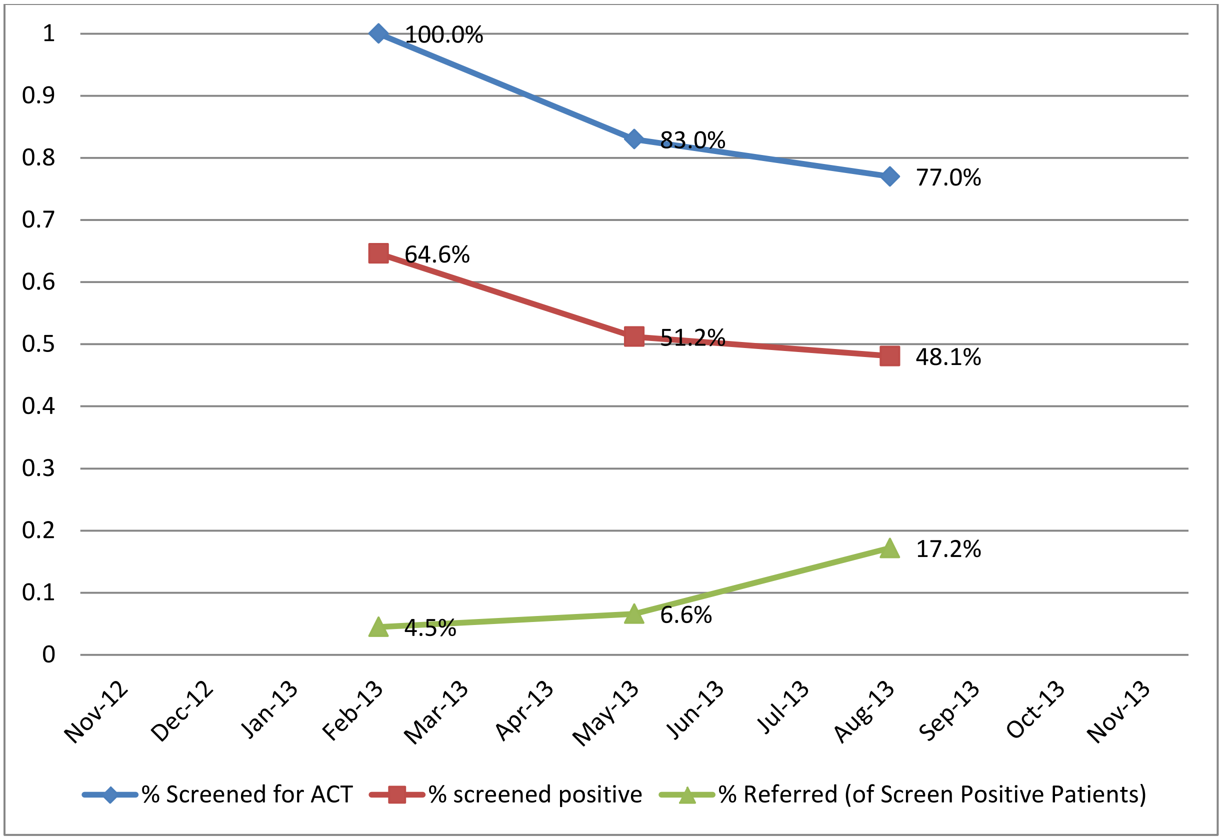

Implementation Events

Poor documentation of referral rates during Q1 and Q2
Implemented EMR changes around Jul 2013 around referral documentation

Q3 Discussion with ACT Clinic

1. Slight decrease in screening rates
2. Substantial increase in referral rates
3. Impact of change in definition of BMI screen positive after Q1
4. Substantial increase in traditional referral and counseling by primary care physician
5. Decrease in self or staff referral

Q4 Discussion 1/15/2014

1. 373 referred per Reach Reporter but tracking sheet indicates only 167 with outreach and 9 self/staff referral – something missing here?
2. No warm handoffs or traditional referrals this quarter – is this what you expected?
3. Counseling rates dropped dramatically - especially for physician counseling, but health coach and BHC rates increased relative to others
4. Primary care clinician counseling in Q3 was really high
