# Supplementary material for: Learning Evaluation: blending quality improvement and implementation research methods to study healthcare innovations
Source: Implement Sci. 2015 Mar 10;10:31. doi: 10.1186/s13012-015-0219-z (PMC4357215; doi:10.1186/s13012-015-0219-z)
Supplement: Additional file 7: — ACT intervention typology. [file 13012_2015_219_MOESM7_ESM.pdf]

Appendix G - Types of Integration Models observed in ACT

	**Bidirectional**	**Behavioral Health in Primary Care**								**Primary Care in Behavioral Health**	
**Characteristics / Practice ID**	**1**	**2**	**3**	**4**	**5**	**6**	**7**	**8**	**9**	**10**	**11**
**Breadth and depth of Integration Reach**											
Identification of Problem	Systematic screening	Systematic Screening	Systematic Screening	Systematic Screening	Clinician Discretion	Clinician Discretion	Clinician Discretion	Clinician Discretion	Systematic Screening	Systematic Screening	Clinician Discretion
Provision of Services	Limited	Broad	Limited	Limited	Limited	Limited	Limited	Limited	Limited	Limited	Limited
**Relevant Workforce Located in Practice**											
Primary care team in practice	Yes	Yes	Yes	Yes	Yes	Yes	Yes	Yes	Yes	Yes	Yes
Embedded BHC on primary care team	Yes	Yes	No	No	Yes	Yes	No	Yes	Yes	No	No
Consulting Psychiatrist in practice	Yes	No	No	No	No	No	In system	In system	In system	In system	No*
**Approach to transitioning patients to BHC**											
Warm hand-off		X				X		X			
Referral	X		X	X	X		X		X	X	X
**Path identified for Other Services**											
Specialty MH	co-located	Referral	Referral	No Path	Referral	Referral	In system	In system	In system	In house	co-located
Substance Use	In house	Referral	Referral	Limited	Referral	Referral	In system	In system	In system	In house	co-located
**Shared Mental Model for Integration**											
Yes		X					X			X	X
No	X		X	X	X	X		X	X		
**Improvements in patient outcomes**											
Yes											
No											

* Use medication management experts

BHC - Behavioral Health Clinician

MH - Mental health
